# Thermodynamics of Micelle Formation of Selected Homologous 7-Alkyl Derivatives of Na-Cholate in Aqueous Solution: Steroid Skeleton and the Alkyl Chain Conformation

**DOI:** 10.3390/ijms252313055

**Published:** 2024-12-04

**Authors:** Dileep Kumar, Mihalj Poša

**Affiliations:** 1 Laboratory for Chemical Computation and Modeling, Institute for Computational Science and Artificial Intelligence, Van Lang University, Ho Chi Minh City 70000, Vietnam; 2 Faculty of Applied Technology, School of Technology, Van Lang University, Ho Chi Minh City 70000, Vietnam; 3 Department of Pharmacy, Faculty of Medicine, University of Novi Sad, Hajduk Veljka 3, 21000 Novi Sad, Serbia

**Keywords:** critical micellar concentration, change in heat capacity of demicellization, steroid skeleton, conformational analysis

## Abstract

Bile acid salts are steroid biosurfactants that build relatively small micelles compared to surfactants with an alkyl chain due to the rigid conformation of the steroid skeleton. In order to increase the capacity of micellar solubilization of the hydrophobic molecular guest, certain C7 alkyl derivatives were synthesized. Namely, introducing an alkyl group in the C7 position of the steroid skeleton results in a more effective increase in the micelle’s hydrophobic domain (core) than the introduction in the C3 position. In comparison, fewer synthetic steps are required than if alkyl groups are introduced into the C12 position of cholic acid in the Grignard reaction. Here, the thermodynamic parameters of micellization (demicellization) of C7 alkyl (number of C atoms in the alkyl group: 2, 3, 4, and 8) derivatives of cholic acid anion in an aqueous solution without additives are examined (which have not yet been determined) in the temperature interval *T* (10–40) °C. The critical micellar concentration and the change in the standard molar enthalpy of demicellization ($∆h_{demic}^{0}$) are determined by isothermal calorimetric titration (ICT). From the temperature dependence of $∆h_{demic}^{0}$, the change in the standard molar heat capacity of demicellization is obtained ($∆C_{demic}^{0}$), the value of which is proportional to the hydrophobic surface of the monomer, which in the micellar state is protected from hydrophobic hydration. The values of $∆C_{demic}^{0}$ indicate that in the case of C7-alkyl derivatives of cholic acid anion with butyl and octyl chains, parts of the steroid skeleton and alkyl chain remain shielded from hydration after disintegration of the micelle. Conformational analysis can show that starting from the C7 butyl chain in the alkyl chain, sequences with gauche conformation are also possible without the formation of steric repulsive strain between the alkyl chain and the steroid skeleton so that the C7 alkyl chain takes an orientation above the convex surface of the steroid skeleton instead of an elongated conformation toward the aqueous solution. This is a significant observation, namely, if the micelle is used as a carrier of a hydrophobic drug and after the breakdown of the micelle in the biological system, the released drug has a lower tendency to associate with the monomer if its hydrophobic surface is smaller, i.e., the alkyl chain is oriented towards the angular methyl groups of the steroid skeleton (the ideal monomer increases the hydrophobic domain of the micelle, but in aqueous solution, it adopts a conformation with the as small hydrophobic surface as possible oriented towards the aqueous solution)—which then does not disturb the passage of the drug through the cell membrane.

## 1. Introduction

Bile acid salts are steroid biosurfactants produced in vertebrates’ livers, known as primary bile acids (cholic and chenodeoxycholic acid). They undergo chemical modifications in the intestinal bacterial flora, producing secondary bile acids (lithocholic and deoxycholic acid) [1,2,3]. When combined with phospholipids, bile acid anions form mixed micelles, which help solubilize cholesterol in the bile canaliculus. In the small intestine, these bile acid salts and their micelles play a crucial role in emulsifying and solubilizing lipids, fat-soluble vitamins, drugs, and so on [3,4,5,6,7,8].

**Figure 1:**
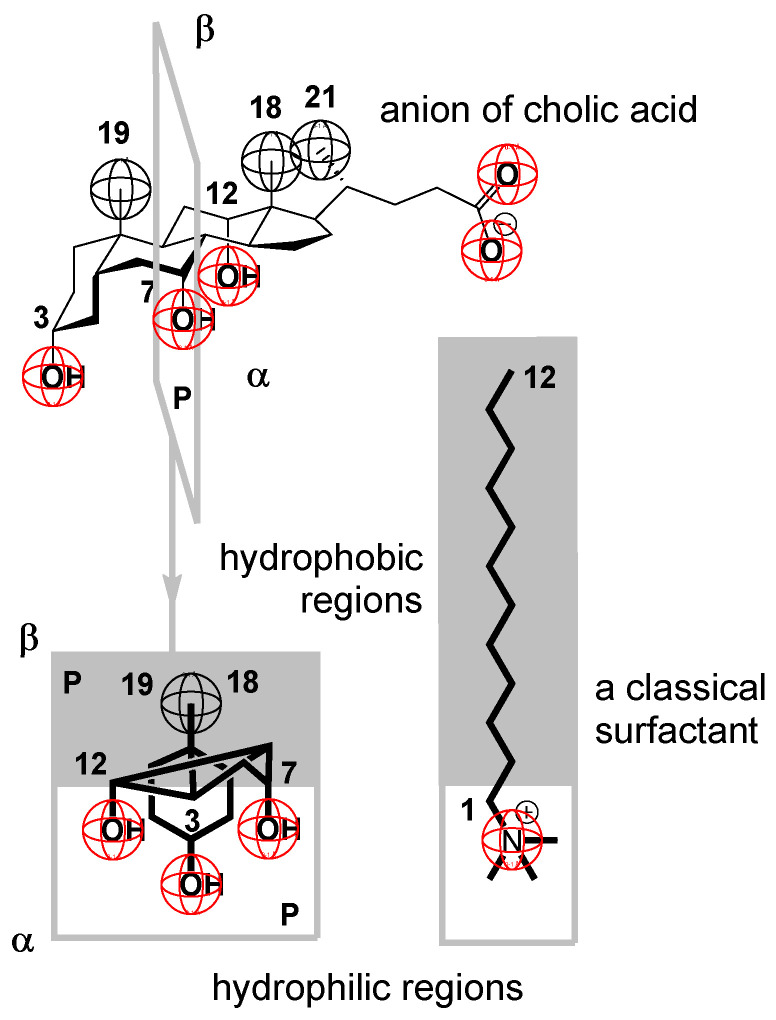
The steroidal skeleton of the cholic acid anion makes this biosurfactant a rigid conformation compared to classical surfactants with an alkyl chain.

Bile acids have a structure dominated by a rigid cyclopentanoperhydrophenanthrene ring with a hydrophobic convex surface (β side of the steroid skeleton) and with a hydrophilic concave surface (α side of the steroid skeleton) [9] (Figure 1). Bile acid anions form relatively small micelles with varying numbers of building units (ranging from 2 to 13) in a water solution [1,4,10,11,12,13]. The bile acid anions in these micelles are oriented over the steroid skeleton’s convex surface (β side), forming primary micelles with hydrophobic interactions [4,14,15,16,17,18]. At higher concentrations, derivatives of bile acid anions in the primary micelles bond over hydrogen bonds, forming larger aggregates known as secondary micelles, according to the Smalls’ concept [4,10,11]. There is ongoing scientific discussion regarding hydrogen bonds in primary micelles [19]. However, molecular dynamic simulations seem to support Smalls’ concept of primary and secondary micelles, acknowledging the possibility of some hydrogen bonds in primary micelles [20,21,22].

The use of bile salts in pharmacy and biomedicine is based on two fundamental properties:

First, their oldest application involves the formation of micelles, which allows bile salts to be used in pharmaceutical formulations. This property helps increase the solubility of hydrophobic drugs in aqueous systems through micellar solubilization. Additionally, bile salts possess detergent properties that can alter the permeability of cell membranes. By forming mixed micelles with phospholipids, they work synergistically to enhance the passive transport of certain drugs across the cell membrane. However, this synergistic effect can also lead to increased membrane toxicity (by pulling out phospholipids, holes are formed in the membrane) for hydrophobic anions of bile acids, thereby raising the hemolytic potential [23,24,25,26,27,28,29,30,31,32,33].

Second, bile acid anions can be applied in their non-micellar or monomeric state. This application is based on the fact that certain bile acid anions act as modulators of specific receptors or enzymes involved in sugar and fat metabolism and the immune response [34,35,36,37,38,39].

To use bile salts as effectively as possible in pharmaceutical formulations, the goal is to increase the hydrophobic surface at the β side of the steroid skeleton. This enhancement improves the solubilization capacity during micellar solubilization of hydrophobic drugs; however, it is also essential to minimize membrane toxicity. The hydrophobic surface area of bile acid anions can be increased by attaching a hydrocarbon chain to the steroid skeleton. This modification can be achieved at the hydroxyl group positions of cholic acid, specifically C3, C7, and C12. Introducing an alkyl chain at either the C7 or C12 position (in the β orientation) effectively increases the hydrophobic surface between the two angular methyl groups, which lies in the central part of the steroid skeleton that is least exposed to hydrophobic hydration in the micellar state.

In contrast, adding an alkyl group at the C3 position is less effective in increasing the hydrophobic surface, as this position in the micellar state is more exposed to hydrophobic hydration. The A ring (containing the C3 OH group) is cis-linked to the B ring (which contains the C7 OH group), causing the alkyl group at C3 to be relatively farther from the angular methyl groups than those at C7 and C12 [5,7,9].

**Figure 2:**
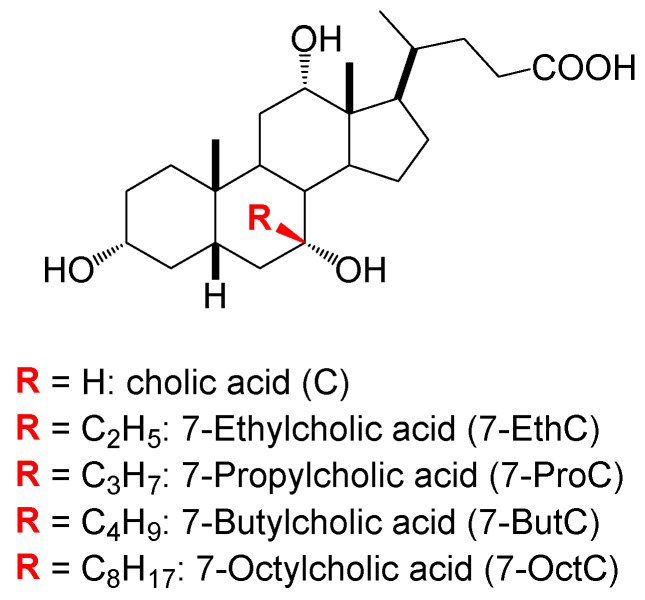
Tested C7-alkyl derivatives of 5β-cholic acid, when determining the thermodynamic parameters of micellization, their Na salts are applied.

The initial reaction when introducing alkyl groups involves oxidizing the steroid’s hydroxyl group. The C7 OH group can be oxidized regioselectively, while the C12 OH group cannot; oxidizing the C12 OH requires protecting the other two hydroxyl groups of cholic acid. Therefore, introducing an alkyl group at the C7 position involves one less reaction step than at the C12 position. Starting with the 7-oxo derivatives of deoxycholic acid (derived from cholic acid by regioselectively oxidizing the C7 OH group), Grignard’s reaction synthesized β7-alkyl derivatives of cholic acid (Figure 2). Similarly, an alkylidene group can be introduced through a Wittig reaction [40,41,42].

Herein the goal is to determine how the C7 alkyl group of Na-cholate affects the self-association process in aqueous solution, i.e., on the thermodynamic parameters of micelle formation. Thus far, the thermodynamic functions of micellization have not been determined for the investigated C7 alkyl derivatives of the anion of cholic acid (Figure 2). The greatest attention is paid to the change in the standard heat capacity of demicellization ($∆C_{demic}^{0}$). Namely, the value of $∆C_{demic}^{0}$ is proportional to the hydrophobic surface of the monomer, which in the micellar state is protected from hydrophobic hydration, and this protected hydrophobic surface of the monomer is hydrated after the disintegration of the micelle [43,44,45,46]. If in a homologous series of surfactants with an increase in the protected hydrophobic surface in the micellar state (in this case, an increase in the C atom of the C7 alkyl chain in the β conformation), there is a deviation from the linear dependence of $∆C_{demic}^{0}$ on the number of C atoms (hydrophobic surface), it means that in the case of monomers, one part of the hydrophobic surface is further protected (as in the micellar state) from hydrophobic hydration, i.e., the alkyl chain in the monomer takes such a conformation that it shields the hydrophobic surface of the steroid skeleton from water molecules [43,44,45,46]. Knowing the available hydrophobic surface of the monomer in the premicellar region (aqueous solution) is essential for pharmaceutical formulations. Whether the drug is incorporated in the micelle and, after the disintegration of the micelle in the biological system, enters into a hydrophobic interaction with the monomers (micellar building units), changing the bioavailability compared to the free drug. The greater the available hydrophobic surface in the bile salt derivative, the more likely its interaction with the hydrophobic drug (below the solubility limit in the premicellar region). Special attention is paid to the conformational analysis of the steroid skeleton with the C7 alkyl chain and the correlation of the conformational states with the thermodynamic functions of micellization.

## 2. Results and Discussion

### 2.1. Theory

Usually, in the literature, the formation of micelles of bile salts is described with a pseudo-phase separation model [43,44,45,46,47,48], according to which if the concentration of monomeric surfactant in an aqueous solution corresponds to the critical micellar concentration (cmc) [49,50,51] (the critical micelle concentration is the total concentration of surfactants at which the aqueous solution/air interface becomes saturated with surfactant molecules; micelles are formed when the total concentration of surfactants exceeds the cmc; at this point, the cmc represents a stable concentration of monomers, while the additional surfactants above the total concentration contribute to the formation of micelles), then monomeric surfactants (bile salts) form a separate micellar pseudo-phase (it is more of a hypothetical phase that unites all micellar particles from an aqueous solution—For the sake of applying the thermodynamic model of the equality of chemical potentials of particles between different phases). In the state of equilibrium, the chemical potential of surfactant *j* in the aqueous solution ($\mu_{\left(aq\right)}$) and the micellar pseudo-phase ($\mu_{M}^{*}$) is equalized. If the chemical potential of surfactant *j* in an aqueous solution is expressed by Henry’s law (ideally diluted aqueous solutions) [52] and if the equilibrium is observed at cmc, then it is expressed as follows:

(1) $$\mu_{M}^{*}=\underset{\mu_{\left(aq\right)}}{\underbrace{\mu_{\left(aq\right)}^{0}+RT\ln x_{j}}}=\mu_{\left(aq\right)}^{0}+RT\ln cmc$$

where $\mu_{\left(aq\right)}^{0}$ represents the standard chemical potential, i.e., the chemical potential of a monomeric surfactant in an infinitely (ideally) diluted aqueous solution, and $x_{j}$ is the mole fraction of surfactant j in the aqueous solution at which the formation of the micellar pseudo-phase begins, which means that in Equation (1) the cmc of surfactant j (bile acid anion) is expressed in mole fraction (*R* and *T* have the usual meaning: universal gas constant and thermodynamic temperature). The change in molar Gibbs energy during the transfer of 1 mole of surfactant j from an infinitely diluted aqueous solution to the micellar pseudo-phase (*p*, *T* = *const.*), i.e., the standard molar Gibbs energy of micellar pseudo-phase formation, is expressed as follows:

(2) $$∆g_{M}^{0}=\mu_{M}^{*}-\mu_{\left(aq\right)}^{0}=RT\ln cmc$$

Since cmc is expressed in mole fraction, $∆g_{M}^{0}<0$ always holds. In isothermal calorimetric titration (ICT), a micellar solution (in order to obtain the most accurate enthalpogram (and therefore the enthalpy of demicellization and cmc value), the criterion is that the micellar solution has a concentration that is at least higher than 10 cmc; usually, if the solubility of the surfactant allows, then 20 cmc is used—before the ICT experiment, the cmc values are known from earlier preliminary experiments) of a surfactant is added in appropriate aliquots to a reaction vessel that initially contains an aqueous solution without surfactant. In the reaction vessel, the surfactant concentration gradually increases; in the initial phase of the titration, it is lower than the cmc value and the surfactant concentration reaches and exceeds the cmc (with each added aliquot of the micellar solution). An energy change is recorded in the reaction vessel, and with each addition of an aliquot of constant concentration, the concentration of surfactant grows in the reaction vessel. In the ICT experiment, if the total surfactant concentration in the reaction vessel is lower than the cmc value, the disintegration of the micellar pseudo-phase will occur (demicellization process) [43,44,45,46,53,54,55], i.e., there is a transfer of the surfactant from the micellar state to the monomer state (in the aqueous solution):

(3) $$-∆g_{M}^{0}=-\left(\mu_{M}^{*}-\mu_{\left(aq\right)}^{0}\right)=\mu_{\left(aq\right)}^{0}-\mu_{M}^{*}=∆g_{demic}^{0}=-RT\ln cmc$$

It follows from Equations (1) and (3) that $∆g_{demic}^{0}>0$. However, since the micelle disintegrates spontaneously when the total surfactant concentration in the aqueous solution is lower than the cmc value (during the ICT experiment), the change in the standard molar Gibbs free energy of demicellization must be negative. In addition to the term $∆g_{demic}^{0}>0$, another term is negative, and the absolute value below the cmc is more significant than $∆g_{demic}^{0}>0$. It is the standard change in the molar Gibbs energy of mixing molecules of monomeric surfactant j and water molecules (and other additives, if any):

(4) $$0>∆g_{mix}^{0}=RTx_{j}\ln x_{j}+RT\sum_{i}x_{i}\ln x_{i}$$

where $x_{i}$ is the mole fraction of water and other additives in the aqueous solution (reaction vessel). Therefore, in the areas of surfactant concentration below the cmc value, the following applies:

(5) $$\left|∆g_{mix}^{0}\right|>∆g_{demic}^{0}$$

The pseudo-phase separation model of micelle formation is analogous to the process when the concentration of a hydrophobic solubilizate gradually increases. When solubility is reached, the appearance of solid phase formation begins, i.e., sediment. The phase separation model does not contain information about the aggregation number (the number of surfactant monomer units in the micellar particle) nor the size of the fraction of counter ions that eventually bind to the micelle [43,44,45,46].

The formation of the micelle $M^{-\left\lceil \nu_{j}z_{j}-\nu_{k}z_{k}\right\rceil }$ of the anionic surfactant $j^{z_{j}-}$ (whose charge is $z_{j^{-}}$) with the aggregation number $\nu_{j^{z_{j}-}}$ can also be viewed as an associative chemical reaction in which the $\nu_{k^{z_{k}+}}$ amount of counterions $k^{z_{k}+}$ is attached to the micelle [56,57]:

(6) $$\nu_{j^{z_{j}-}}j^{z_{j}-}+\nu_{k^{z_{k}+}}k^{z_{k}+}\to M^{-\left[\nu_{j}z_{j}-\nu_{k}z_{k}\right]}$$

If no counterions are added to the surfactant aqueous solution, the counterions come from the salt from which the anionic surfactant comes. Then, the charge of the micelle remains negative, i.e., $-\left[\nu_{j}z_{j}-\nu_{k}z_{k}\right]$ [58]. At *p*, *T* = *const.*, the change in molar Gibbs free energy during the differential progress (dξ) of reaction (6) is $dg=\sum_{i}\nu_{i}\mu_{i}d\xi$. By applying chemical potentials for ideally diluted aqueous solutions in a state of equilibrium (${\left(\partial G/\partial \xi \right)}_{p,T}=0$), the following expression holds:

(7) $$∆g_{M}^{0}=\left(\mu_{M^{-\left[\nu_{j}z_{j}-\nu_{k}z_{k}\right]}}^{0}-\nu_{j^{z_{j}-}}\mu_{j^{z_{j}-}}^{0}-{\nu_{k^{z_{k}+}}\mu }_{k^{z_{k}+}}^{0}\right)/\nu_{j^{z_{j}-}}=-RT\ln x_{M^{-\left[\nu_{j}z_{j}-\nu_{k}z_{k}\right]}}^{1/\nu_{j^{z_{j}-}}}+RT\ln x_{j^{z_{j}-}}+RT(\nu_{k^{z_{k}+}}/\nu_{j^{z_{j}-}})\ln x_{k^{z_{k}+}}$$

In the case of bile salts, $\left|z_{j^{-}}\right|=z_{k^{+}}=1$, and if the balance of micellization is observed at cmc values, at which the amount of surfactant (bile acid anion) that forms the micelle can be ignored (at cmc values, only a few micellar particles are formed) so that the mole fraction of counterions is equal with cmc, and if there is a limit value:

(8) $$\underset{\nu_{j^{-}}\to \infty }{\lim}\ln x_{M^{-\left[\nu_{j}-\nu_{k}\right]}}^{1/\nu_{j^{-}}}=0$$

Then, Equation (7) is:

(9) $$∆g_{M}^{0}=\left(\mu_{M^{-\left[\nu_{j}-\nu_{k}\right]}}^{0}-\nu_{j^{-}}\mu_{j^{-}}^{0}-{\nu_{k^{+}}\mu }_{k^{+}}^{0}\right)/\nu_{j^{-}}=\left(1+\beta \right)RT\ln cmc=\left(2-\vartheta \right)RT\ln cmc$$

where *β* represents the fraction of bile acid anion charge from the micellar particle that is neutralized with the counterion charge ($\beta =\nu_{k^{+}}/\nu_{j^{-}}$) [58]. Parmeter ϑ=1−β represents the degree of counterion dissociation from the micelle particle [59]. There are a certain number of bile salt derivatives (i.e., their micelles) where, based on conductometric measurements, β can be neglected (ϑ=1; or 100% dissociation of the counterion from micelle), then Equation (8) is equal to the equation obtained based on the pseudo-phase separation model (2):

(10) $${\left(∆g_{M}^{0}\right)}_{association}=RT\ln cmc={\left(∆g_{M}^{0}\right)}_{pseudo-phaseseparation}$$

Of course, the equality holds in case the limit value (8) exists, which is acceptable for surfactants with a relatively long alkyl hydrophobic chain (aggregation number from 40 to 200). Bile acid anions build relatively small micelles, compared to classic surfactants, with a few micellar building units (from 4 to 13 in Small’s primary micelles) [10,11]. Therefore, Equality (10) should be:

(11) $${\left(∆g_{M}^{0}\right)}_{association}=-RT(1/\nu_{j^{-}})\ln x_{M^{-\left\lceil \nu_{j}-\nu_{k}\right\rceil }}+{\left(∆g_{M}^{0}\right)}_{pseudo-phaseseparation}$$

(12) $${\left(∆g_{M}^{0}\right)}_{pseudo-phaseseparation}={\left(∆g_{M}^{0}\right)}_{association}+RT(1/\nu_{j^{-}})\ln x_{M^{-\left\lceil \nu_{j}-\nu_{k}\right\rceil }}$$

However, as the equilibrium concentration, i.e., micelle mole fraction ($x_{M^{-\left\lceil \nu_{j}-\nu_{k}\right\rceil }}$) mainly depends on the degree of hydrophobicity of the convex, C7, and C12 lateral surfaces of the steroid skeleton (the higher the hydrophobicity of the steroid skeleton, the greater the equilibrium constant for micelle formation, leading to a higher mole fraction of the micelle.), then the term $-RT(1/\nu_{j^{-}})\ln x_{M^{-\left\lceil \nu_{j}-\nu_{k}\right\rceil }}$ can be viewed as an error that depends on the hydrophobicity of the steroid skeleton [9,10,11], similar to how cmc depends on the hydrophobicity of the β side of the steroid skeleton (i.e., term RTln⁡cmc) [60,61,62,63,64]:

(13) $${\left(∆g_{M}^{0}\right)}_{pseudo-phaseseparation}={\left(∆g_{M}^{0}\right)}_{association}+{\left(∆g_{M}^{0}\right)}_{error}$$

Therefore, in Equation (13), both terms are functions of the hydrophobic surface of the steroid skeleton (${\left(∆g_{M}^{0}\right)}_{error}=f\left(hydrophobicsurface\right)$ and ${\left(∆g_{M}^{0}\right)}_{association}=f\left(hydrophobicsurface\right)$), so that ${\left(∆g_{M}^{0}\right)}_{pseudo-phaseseparation}$, which is itself a function of the hydrophobic surface on the β side of the steroid skeleton, although loaded with error, correctly reflects the structural differences in the steroid skeleton of bile acid anions. Consequently, many authors use the pseudo-phase separation model to describe the thermodynamic parameters of the micellization of bile salts. By applying the pseudo-phase separation method, not only can bile acid anions be compared according to the hydrophobicity of the steroid skeleton (contained in the thermodynamic functions of micelle formation), but at the same time, $∆g_{M}^{0}$ values can be compared with literature values (where the phase separation method was also applied) [43,44,45,46].

### 2.2. Results

ICT experiments are conducted in aqueous solutions. The acid dissociation constant (*K*_a_) of bile acids is on the order of 10^−5^, which leads to their hydrolysis constant (*K*_h_) being approximately 10^−9^ (*K*_h_ = *K*_w_/*K*_a_, where *K*_w_ = 10^−14^). The degree of hydrolysis (*l*), which represents the proportion of bile acid anions that become protonated due to their reaction with water, is calculated using the following quadratic equation:

(14) $$l^{2}c_{T}+lK_{h}-K_{h}=0$$

Here, $c_{T}$ refers to the total concentration of the conjugated base, or bile acid salt. When $c_{T}$ equals the critical micelle concentration (cmc), the degree of hydrolysis (*l*) is found to be less than 0.5%. This small fraction of undissociated species has a negligible impact on the cmc value. While bile acid anion micelles may solubilize undissociated acid as acid–salt mixed micelles, the amount of solubilization can be considered insignificant [12,13,19].

In the ICT experiment at a certain constant temperature, the cmc value and the change in the standard molar enthalpy of demicellization ($∆h_{demic}^{0}$) are obtained in the same experiment. The change in the standard molar Gibbs free energy of demicellization is obtained from the cmc value using Equation (2) or Equation (9). The change in the standard molar entropy of demicellization is calculated from the Gibbs–Helmholtz equation: $∆g_{demic}^{0}=∆h_{demic}^{0}-T∆s_{demic}^{0}$ (Table 1).

The critical micellar concentration of cholic acid anion (aqueous solution without additives, Table 1) is in agreement with the literature data obtained in the ICT experiment under identical conditions (10 °C: 13.0 mM; 25 °C: 10.4 mM; 40 °C: 16.5 mM [46]). By introducing an alkyl group into the C7 position of the steroid skeleton of the cholic acid anion, the cmc value at each tested temperature decreases about the cmc value of the cholic acid anion, and more, if the C7 alkyl chain is longer (Table 1). Namely, increasing the length of the C7 alkyl chain increases the overall hydrophobicity of the steroid skeleton [42]. Our earlier investigations in the NMR experiment found that the alkyl chain has β orientation, while the C7 OH group remains in α orientation [40].

**Figure 3:**
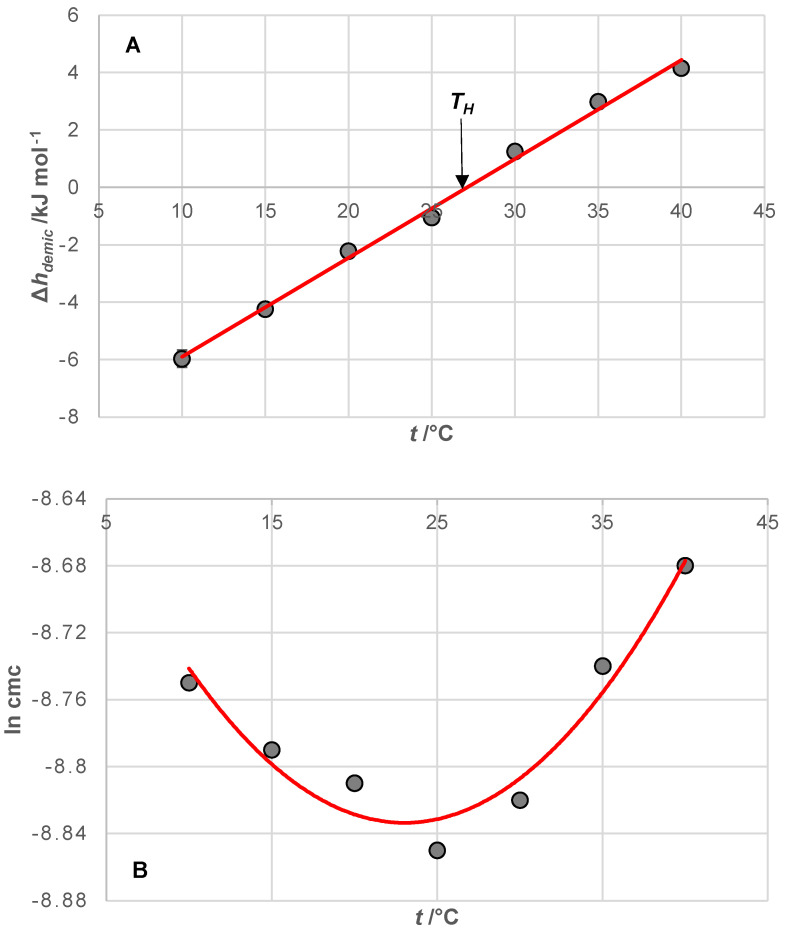
An example of the dependence of the change in the standard molar enthalpy of demicellization (**A**) and the dependence of the logarithm of the cmc value on temperature for 7-OctC (**B**); *T_H_* = 27 °C.

For each investigated bile acid anion, there is a temperature (*T_H_*) at which $∆h_{demic}^{0}$ has a zero value (for the investigated bile acid anions, *T_H_* is around 25 °C, Figure 3A and Table 1). At temperatures lower than *T_H_*, $∆h_{demic}^{0}<0$ applies, while $∆h_{demic}^{0}>0$ applies at temperatures higher than *T_H_*. The literature value of temperature T_H_ for bile acid anion C is 30 °C [44]. Usually, the *T_H_* temperature does not show dependence on the hydrophobicity of the steroid skeleton like cmc. However, the appearance of the *T_H_* temperature is a characteristic of the hydrophobic effect [63,64]. According to van’t Hoff’s equation:

(15) $${\left(\partial \ln cmc/\partial T\right)}_{p}=∆h_{demic}^{0}/\left(T^{2}R\right)$$

The slope of the ln⁡cmc=f(T) function depends on the change in the standard molar enthalpy of demicellization. If $∆h_{demic}^{0}<0$, then the function ln⁡cmc=f(T) decreases with increasing temperature; at temperature T_H_, the function ln⁡cmc=f(T) has a minimum value—the lowest cmc value for the examined bile acid anion, i.e., the temperature at which the examined bile salt is the most hydrophobic—and if $∆h_{demic}^{0}>0$, then the function ln⁡cmc=f(T) increases with increasing temperature (Figure 3).

**Figure 4:**
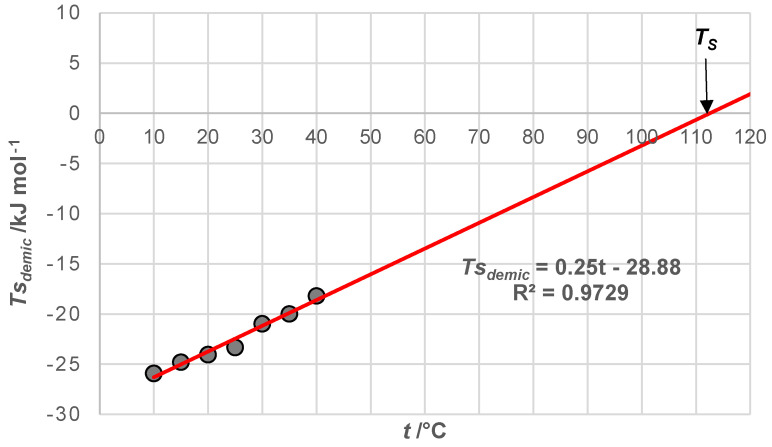
*T_S_* temperature for 7-ButC: micelle formation has an enthalpic driving force.

By extrapolating the linear dependence of $T∆s_{demic}^{0}=f(T)$ to the value$\;T∆s_{demic}^{0}=0$, the temperature *T_S_* is obtained, at which the process of micellization (demicellization) exclusively depends on the enthalpy effect (Figure 4). The literature value of *T_S_* temperature is in the interval (100–140) °C [44,63].

**Figure 5:**
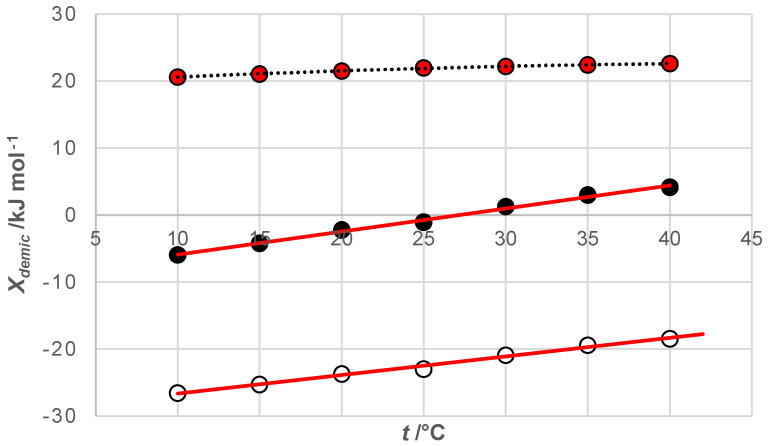
Temperature dependence of thermodynamic potentials of demicellization and entropy of demicellization: X = thermodynamic potentials *g* (dashed curve), *h* (solid line with black circles) and product of temperature and entropy (solid line with empty circles); example for 7-OctC.

For the examined bile acid anions, the temperature dependence of the change in the standard molar Gibbs free energy of demicellization is weak. In contrast, the changes in $∆h_{demic}^{0}$ and $T∆s_{demic}^{0}$ with temperature are more pronounced. Straight lines representing the functions $∆h_{demic}^{0}=f(T)$ and $T∆s_{demic}^{0}=f(T)$ are approximately parallel to each other (Figure 5 and Table 1). This is characteristic of enthalpy–entropy compensation [43,44,45,46,63,64,65].

### 2.3. Discussion

**Figure 6:**
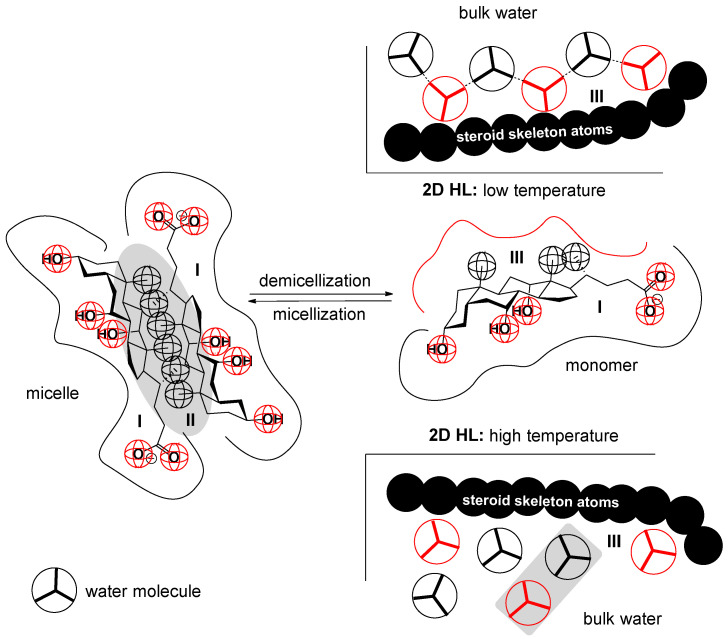
In the micellar state, a hydration layer (I) forms around the polar groups of monomers (micellar building units), which remains unchanged after the disintegration of the micelle. In the micellar state, the hydrophobic surface of the bile acid anion’s steroid skeleton forms the micelle’s hydrophobic core (II) and is protected from hydration. During demicellization, a new hydration layer (III) is formed above the hydrophobic surface of the steroid skeleton. In the hydration layer above the hydrophobic surface at low temperatures, it is true that the water molecules immediately above the atoms of the steroid skeleton are more ordered than the water molecules from the bulk solution (they have lower entropy than the bulk water) and have a coiled orientation for building H-bonds with water molecules from the inside (2D HL = two-dimensional representation of the hydration layer). With increasing temperature, the mobility of water molecules from the hydration layer above the hydrophobic surface of the steroid skeleton increases. The exchange frequency of these water molecules with water molecules from the bulk increases (the entropy of water molecules and the entropy of water molecules from the hydration layer become equal), and these water molecules lose their favorable orientation for building the H-bonds.

The standard molar enthalpy changes associated with demicellization ($∆h_{demic}^{0}$) are negative at temperatures below the threshold temperature (*T* < *T_H_*) (Table 1 and Figure 3). When a bile acid anion (monomer) transitions from the micellar pseudo-phase to the aqueous phase, a hydration layer forms over the monomer’s hydrophobic surface [64,66,67,68]; in this hydration layer, water molecules orient themselves in a manner that optimizes the formation of hydrogen bonds with water molecules from the bulk solution [69,70]. This formation of hydrogen bonds results in a change in enthalpy ($∆h_{H-bonds}^{0}$), where the absolute value of the energy released during hydrogen bond formation exceeds the energy required to break the van der Waals intramolecular secondary bonds (hydrophobic interactions) between the monomers within the micellar pseudo-phase ($∆h_{W-bonds\;in\;M}^{0}$; the hydrophobic domain of the micelle) (Figure 6). As a result, the net change in enthalpy, which represents the standard molar enthalpy changes in demicellization, is negative:

(16) $$0>∆h_{demic}^{0}=∆h_{H-bonds}^{0}+∆h_{W-bondsinM}^{0}$$

As temperature rises, the absolute value of the energy associated with the hydrophobic interactions between the hydrophobic surfaces of the steroid skeletons of the monomers in the interior of the micelle also increases ($∆h_{W-bondsinM}^{0}$). At temperatures above the threshold temperature (*T* > *T_H_*), the heat energy required to break these hydrophobic interactions surpasses the absolute value of the enthalpy change due to the hydration of the monomer’s hydrophobic surface during demicellization ($\left|∆h_{W-bondsinM}^{0}\right|>\left|∆h_{H-bonds}^{0}\right|$). This occurs because the absolute value of $\left|∆h_{H-bonds}^{0}\right|$ decreases with increasing temperature due to the greater mobility of water molecules in the hydration layer, which diminishes the alignment necessary for effective hydrogen bonding [68,69] (Figure 6). Consequently, at temperatures higher than *T_H_*, the standard molar enthalpy change in demicellization becomes positive ($∆h_{demic}^{0}>0$).

At *T_H_*, $∆h_{demic}^{0}$ equals zero, indicating that at this temperature, the hydrophobic effect driving micelle formation results solely from changes in entropy. This entropy change is due to the movement of water molecules from the ordered state in the hydration layer along the hydrophobic surface to the less ordered state in the bulk solution. As the temperature increases, the critical micellar concentration of 7-alkylC derivatives (7-EthC, 7-ProC, 7-ButC, and 7-OctC; Table 1 and Figure 2) also rises. Specifically, with higher temperatures, the hydrophobic effect diminishes because the increased mobility of water molecules in the hydration layer reduces the orderliness around the hydrophobic surface of the monomer, thereby decreasing the entropic effect of self-association [64]. At *T_S_* temperature (Figure 4), the molar entropy of water from the hydration layer above the hydrophobic surface of the steroid skeleton equals the molar entropy of water from the bulk solution. Therefore, the formation of micelles has an exclusively enthalpic driving force (which originates from van der Waals interactions between the micellar building blocks in the micellar pseudo-phase): $T_{S}∆s_{demic}^{0}=0$ and $∆g_{demic}^{0}=∆h_{demic}^{0}$.

**Figure 7:**
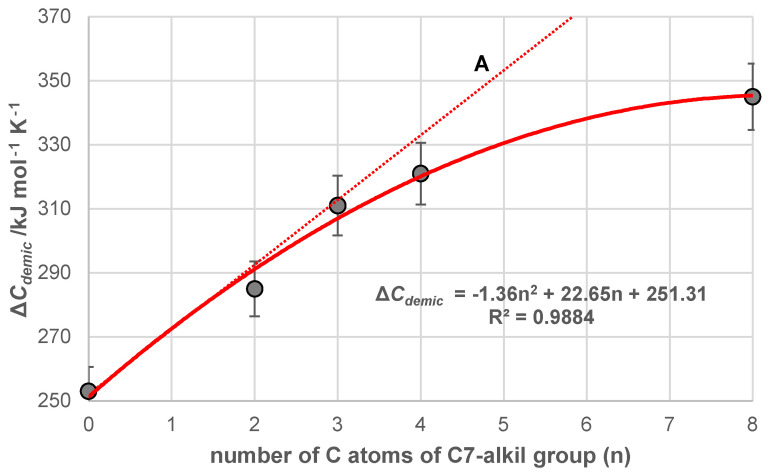
Dependence of the change in the heat capacity of demicellization on the number of carbons of the C7 alkyl chain in the investigated bile salt derivatives.

The change in heat capacity of demicellization $∆C_{demic}^{0}={\left(\partial ∆h_{demic}^{0}/\partial T\right)}_{p}$ is a parameter that is related (i.e., proportional) to the hydrophobic surface of the steroid skeleton of the bile acid anion, which (the surface) is shielded (protected) from hydrophobic hydration in the micelle. During the demicellization process, the shielded hydrophobic surface of the steroid skeleton is hydrated. From the $∆C_{demic}^{0}$ value of the 7-alkylC derivative (7-EthC, 7-ProC, 7-ButC, and 7-OctC; Table 1), it can be concluded that the hydration-protected hydrophobic surface of the micellar building block increases with the length of the C7 alkyl chain. However, the increase in the $∆C_{demic}^{0}$ value with the length of the alkyl chain is not linear. Nevertheless, from the butyl chain, the increases in this parameter slow down (Figure 7), which is probably a consequence of the different conformation of the C7 alkyl chain in 7-ButC and 7-OctC derivatives compared to the conformations in 7-EthC and 7-PropC derivatives.

**Figure 8:**
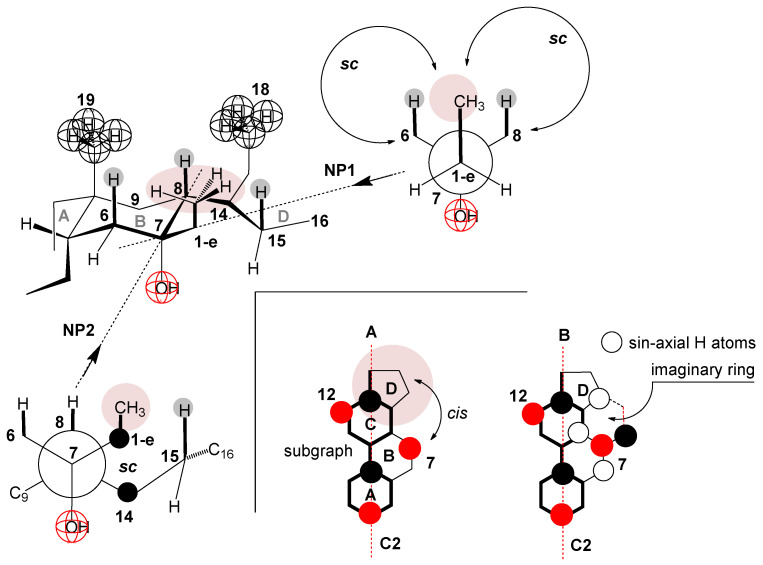
Syn-axial orientation of the methyl group from the C7 ethyl group of the derivative 7-EthC (NP = Newman projection formula and A, B = molecular subgraph).

As the 1-e C atom of the C7 alkyl chain is in the β orientation [40] (Figure 8), the methyl group from the C7 ethyl group (7-EthC derivative) can have an orientation in which this methyl group is less exposed to hydrophobic hydration (find it in the space already screened by the C18 and C19 angular methyl groups and the cis D ring [9], Figure 8A,B) but is in a synclinal (*sc*) relationship with the C6 and C8 axial hydrogen atoms (Newman projection NP1) as well as with C15 pseudo-axial hydrogen (NP2 and Figure 8B)—steric strain arises [71,72,73]. Viewed in the entirety of the steroid skeleton of the 7-EthC derivative, the methyl group of the C7 ethyl group is in syn-axial orientation with the axial hydrogens (Figure 8). This methyl group suffers steric strain but is exposed to hydration to a lesser extent. However, the relatively large value of the change in the heat capacity of demicellization according to the value of the same parameter in derivative C compared to the exact change in 7-ButC and 7OctC derivatives (Figure 7) indicates that the conformation of the C7 ethyl group is such that it is oriented towards the interior of the aqueous solution and not towards the convex side of the steroid skeleton (Figure 8). Therefore, during demicellization, it is exposed to hydrophobic hydration to a greater degree; i.e., in the micelle, this ethyl group is protected from hydrophobic hydration, while in the monomeric state (after the disintegration of the micelle), it is no longer shielded.

**Figure 9:**
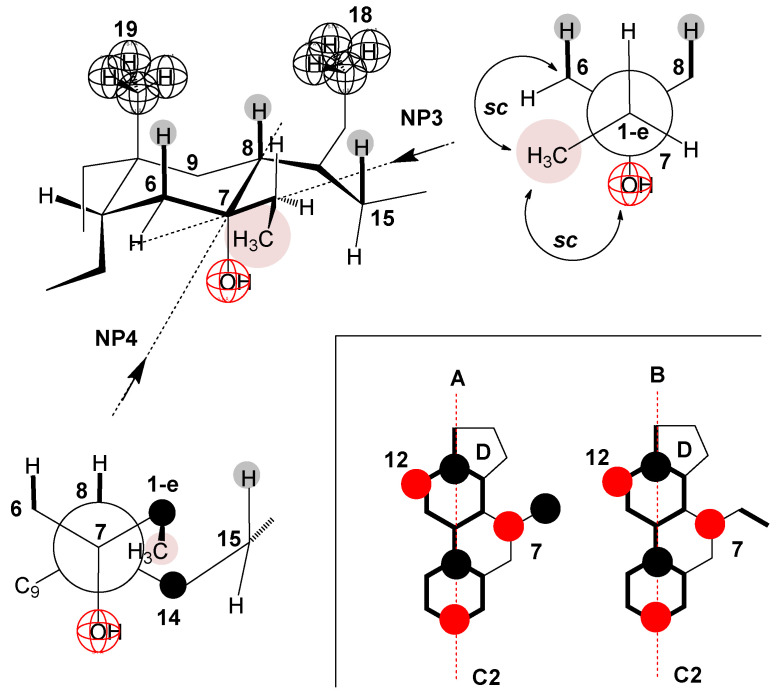
Partial conformation of the steroid skeleton of 7-EthC (NP = Newman projection formula) in which the methyl group from the C7 ethyl group is not in syn-axial orientation (A) with the corresponding axial hydrogens of the steroid skeleton but is oriented towards the interior of the solution (B).

Namely, in the partial conformation represented by the NP3 Newman projection formula (Figure 9), the methyl group from the C7 ethyl group has an *sc* position to the C6 methylene group, which creates particular steric strain (3.8 kJ mol^−^^1^) [71]. However, this steric strain is undoubtedly lower than if this methyl group is in a sternal orientation that is parallel to the axial hydrogen atoms from the steroid skeleton (H atoms from C6, C8, and C15, NP1 and NP2, Figure 8)—where there are three *sc* (gauche) interactions with a steric strain of 11.4 kJ mol^−^^1^. According to NP4 Newman’s projection formula (Figure 9), the methyl group from the C7 ethyl group is *sc* with the C14 methine group of the steroid skeleton; however, with this *sc* orientation, no steric strain is created since the methyl group from the 1-e C atom is oriented towards the interior of the solution and not towards the steroid skeleton, i.e., on the C14 methine group there is only axial hydrogen that is oriented towards the concave surface (α side) of the steroid skeleton and not towards the 1-e C atom. Therefore, there is no spatial element in which two unbonded atoms are simultaneous, so the *sc* mutual position in NP4 does not create sternal repulsion. Therefore, starting from the C8 methine group through the C7 and 1-e carbons to the methyl group (from the C7 ethyl group), there is a partial conformation in which the C8 methine carbon and the methyl group (from the C7 ethyl group) are in an antiperiplanar conformation—i.e., this sequence contains the lowest possible steric strain (NP3 Figure 9). The above-described position of the C7 ethyl group corresponds to the molecular graph B (Figure 9), from which it can be seen that the C7 ethyl group is oriented towards the interior of the aqueous solution and not towards the angular metal groups on the β side of the steroid skeleton (represented by molecular subgraph A, Figure 9), which means that when the micelle breaks up (the state in which the C7 ethyl group is protected from the hydrophobic hydration), in the monomeric state of the 7-EthC derivative, the C7 ethyl group will be exposed to hydration, which increases the change in the standard molar heat capacity of demicellization in relation to the same parameter at C.

**Figure 10:**
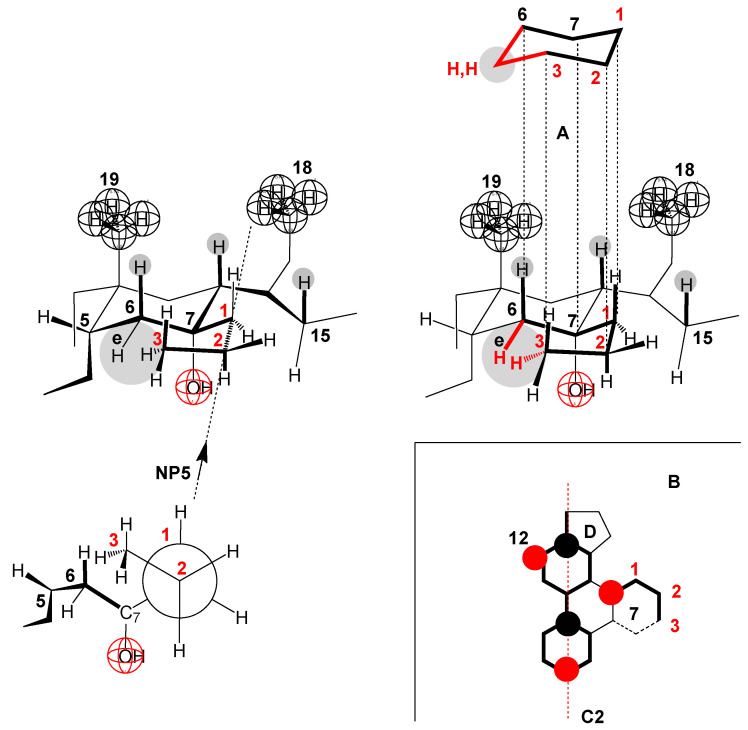
Conformation of the C7 propyl group in 7-PropC derivatives when the propyl hydrocarbon chain is in the gauche conformation: hydrophobic hydration decreases, but steric strain increases (NP = Newman projection formula and A, B = molecular subgraph).

**Figure 11:**
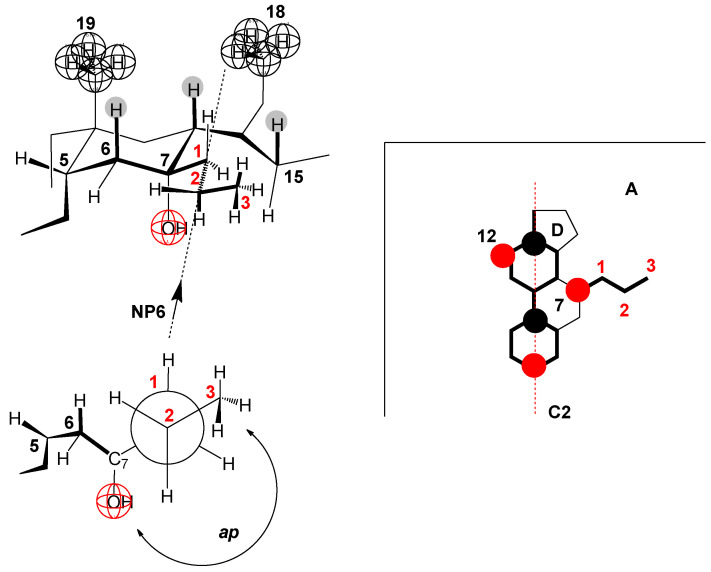
The conformation of the propyl group in which there is no steric strain (the methyl group and the C7 carbon from the steroid skeleton are in an antiperiplanar (*ap*) relationship NP6) but the hydrophobic hydration of the C7 propyl group is maximal (A).

In the 7-ProC derivative, the value of the parameter $∆C_{demic}^{0}$ (Figure 7) indicates that the conformation of the C7 propyl group is elongated. Namely, if the terminal methyl group of the C7 propyl group in the corresponding Newman projection formula is in the *sc* position to the C7 carbon of the steroid skeleton—Gauch conformation—(NP5 Figure 10), then the propyl chain, C6 and C7 carbons of the steroid skeleton would form a chair conformation of a partial cyclohexane’s ring (Figure 10A) where the C6 equatorial hydrogen and one of the hydrogens of the terminal methyl group of the C7 propyl group would be in the same space element, resulting in a significant steric repulsive interaction. However, regarding hydrophobic hydration, part of the C7 propyl group would be protected from forming a hydration cage (the part towards the steroid skeleton (Figure 10B). Suppose the terminal methyl group of the C7 propyl group in the NP6 Neman projection formula (Figure 11) is in the antiperiplanar (*ap*) position to the C7 carbon of the steroid skeleton (elongated conformation of the propyl group). In that case, there is no steric repulsive strain either in the propyl group itself or between the C7 propyl group and the steroid skeleton; however, in the elongated C7 conformation, the propyl group is fully exposed to hydrophobic hydration (it is oriented towards the interior of the solution, Figure 11A), when the 7-ProC monomer is in aqueous solution after micelle disintegration. This results in a significant value of the parameter $∆C_{demic}^{0}$ compared to the same parameter of the reference derivative C.

**Figure 12:**
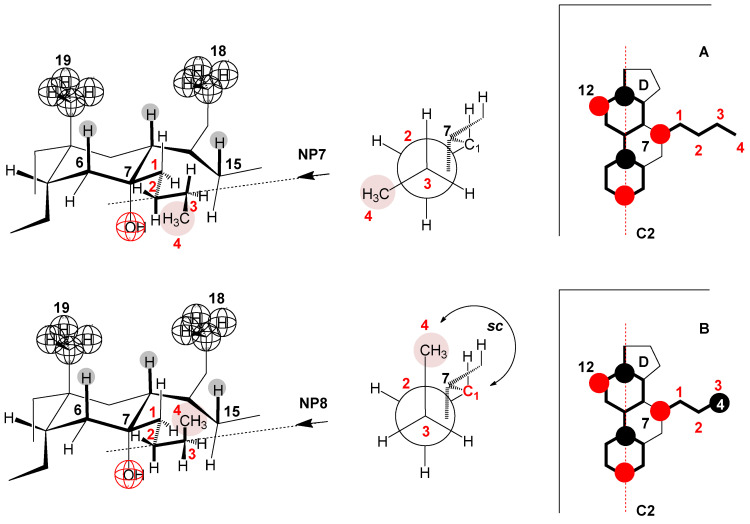
Partial conformations of 7-ButC, with this cholic acid anion derivative, a gauche conformation of the C7 side chain is possible without inducing a steric strain with the steroid skeleton (NP = Newman projection formula and A, B = molecular subgraph).

In the case of the 7-ButC derivative, the deviation from the linear dependence of the change in the standard molar heat capacity on the number of C atoms of the C7 alkyl chain begins (Figure 7A). Namely, in this bile acid anion derivative, in addition to the elongated conformation of the C7-butyl chain (the antiperiplanar relationship of the terminal methyl group from the C7-butyl group and the C1 methylene group of the same alkyl chain in Newman’s projection formula NP7 and the elongated chain in molecular graph A, Figure 12)—without the existence of steric strain—a gauche conformation of the C7 butyl chain is also possible, in which the terminal methyl group of the alkyl chain and the C1 methylene group are in a synclinal mutual position in the NP8 Newman projection formula (Figure 12) with a steric strain of 3.8 kJ mol^−^^1^, but without repulsive steric interactions with the steroid skeleton. Adopting the NP8 gauch conformation by the C7 butyl chain reduces the exposure of this alkyl chain to hydrophobic hydration when the monomer (7-ButC) is in aqueous solution. Especially the terminal methyl group of the butyl chain is sterically shielded since it is parallel to the syn-axial hydrogen atoms of the steroid skeleton, which prevents the access of water molecules (Figure 12 and subgraph B), i.e., prevents the formation of a hydration layer above the part of the hydrophobic surface of the C7 butyl chain.

**Figure 13:**
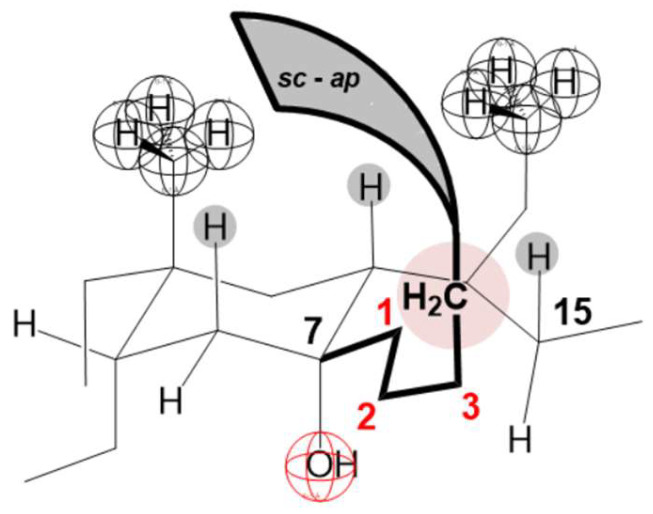
In the case of C7 alkyl derivatives of the anion of cholic acid, if the alkyl chain contains four or more carbons, then the alkyl chain in partial gauche (synclinal, *sc*) and antiperiplanar (*ap*) conformations occupies the space above the convex surface of the steroid skeleton, which reduces the hydrophobic hydration.

The change in the standard molar heat capacity of demicellization of 7-OctC significantly deviates from line A (Figure 7), which indicates that in this derivative, after the micelle disintegration, part of the alkyl (octyl) chain remains protected from hydrophobic hydration. Namely, the octyl chain after the first of the three C atoms can have partial chains of C atoms in the gauche conformation without generating steric strain with the steroid skeleton, which means that the octyl chain in space can occupy a position (conformation) above the convex surface (β side [74,75]) of the steroid skeleton so that the space between the part of the steroid skeleton and the octyl chain is protected from hydrophobic hydration (Figure 13). If there were no twisting of the octyl chain towards the angular methyl groups of the steroid skeleton, it would have an elongated conformation towards the interior of the aqueous solution. In the case of the 7-OctC derivative, the value of the change in the standard molar heat capacity of demicellization would be on line A (Figure 7).

In the analyzed 7-alkyl derivatives of cholic acid anions, the dissociation of counterions from micellar particles ranges from 90% to 95% (Table 2). This implies that the bile acid anions in these micellar particles behave as if they were in a monomeric state. Precisely, the steroid C17 side chains with carboxylate functions of adjacent micellar building units are relatively spaced apart, preventing the formation of a Stern layer where the counterion would typically be located (bound). Our earlier research indicates that in the examined C7 alkyl derivatives of cholic acid anions, the aggregation number, regardless of the alkyl chain length, falls between 9 and 12 building units [42]. However, derivatives with longer alkyl chains demonstrate a greater capacity to solubilize hydrophobic substances in aqueous solutions. This suggests that during the formation of mixed micelles with hydrophobic solubilizates, a certain number of C7 octyl derivatives of cholic acid are present in the micellar phase, where the C7 octyl group adopts an elongated conformation.

## 3. Materials and Methods

The 3α,12α-dihydroxy-7-oxo-5β-cholanoic acid (7-oxodeoxycholic acid) was obtained according to Tullar from the cholic acid [76]. Synthesis (from 7-oxodeoxycholic acid) and chemical characterizations of 7-EthC, 7-ProC, 7-ButC, and 7-OctC have been published previously [40,42]. The cholic acid (Sigma, Auckland, New Zealand; purity ≥ 99%) was used as received. All bile acids were transformed into sodium salts by a known procedure [7].

**Figure 14:**
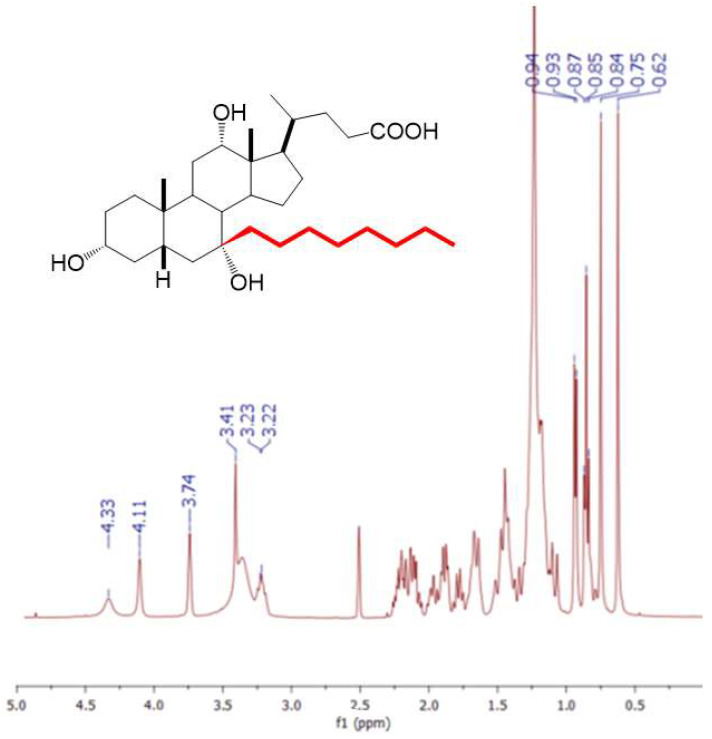
^1^H NMR (400 MHz, DMSO-*d*_6_) of 7-OctC.

Detailed structural characterization and preliminary investigations of 7-alkyl cholic acid derivatives are given in our earlier works [40,42]. 7-EtC: ^1^H NMR (400 MHz, DMSO-*d*_6_) δ = 0.64 (s, 3H, H-18), 0.76 (s, 3H, H-19), 0.79 (t, *J* = 7 Hz, 3H, CH_3_ ethyl chain), 0.94 (d, *J* = 6 Hz, 3H, H-21), 3.22 (m, 1H, H-3), 3.41 (s, 1H, OH on H-7), 3.75 (s, 1H, H-12), 4.10 (s, 1H, OH on C-12), 4.33 (s, 1H, OH on C-3). 7-ProC: ^1^H NMR (400 MHz, DMSO-*d*_6_) δ = 0.66 (s, 3H, H-18), 0.79 (s, 3H, H-19), 0.83 (t, *J* = 7 Hz, 3H, CH_3_ propyl chain), 0.98 (d, *J* = 6 Hz, 3H, H-21), 3.22 (m, 1H, H-3), 3.41 (s, 1H, OH on H-7), 3.75 (s, 1H, H-12), 4.10 (s, 1H, OH on C-12), 4.33 (s, 1H, OH on C-3). 7-ButC: ^1^H NMR (400 MHz, DMSO-*d*_6_) δ = 0.75 (s, 3H, H-18), 0.85 (s, 3H, H-19), 0.92 (t, *J* = 7 Hz, 3H, CH_3_ butyl chain), 1.03 (d, *J* = 6 Hz, 3H, H-21), 3.54 (m, 1H, H-3), 3.95 (s, 1H, H-12). 7-OctC: ^1^H NMR (400 MHz, DMSO-*d*_6_) δ = 0.62 (s, 3H, H-18), 0.75 (s, 3H, H-19), 0.85 (t, *J* = 7 Hz, 3H, CH_3_ octyl chain), 0.93 (d, *J* = 6 Hz, 3H, H-21), 3.22 (m, 1H, H-3), 3.41 (s, 1H, OH on C-7), 3.74 (s, 1H, H-12), 4.11 (s, 1H, OH on C-12), 4.33 (s, 1H, OH on C-3) (Figure 14).

**Figure 15:**
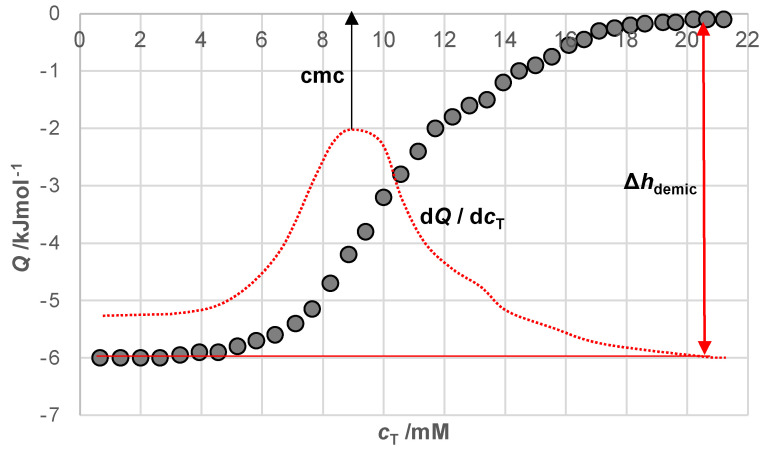
Reaction enthalpy (*Q*) vs. the total detergent concentration in the reaction cell (*C*_T_); titration of 135 mM 7-OctC in water into 2 mL water at 10 °C (37 injections of 10 μL aliquots).

Thermometric titration experiments [19] were performed at temperatures of 10, 15, 20, 25, 30, 35, 4and 0 °C with a thermal activity monitor isothermal heat-flow microcalorimeter (ThermoMetric LKB 2277, Lund, Sweden) and a twin detector supplied with a sample cell and a reference cell. The sample cell, equipped with a stirring facility and a Lund microtitrator, was loaded with 2 mL of water. A stirring rate of 60 rpm was applied, and titrant (0.5 mL of bile salts solution in water at ≈15 cmc) was injected into the cell at 90-min intervals in aliquots of 10 μL (Figure 15). The experiment was computer-controlled via DigiTam 4.1 software. The noise level of the calorimeter baseline during measurements was within ±0.05 μJ s^−1^. On average, the reproducibility of calorimetric peaks in the titration experiment was better than 5%.

The critical micellar concentration (cmc) is obtained based on the first derivative of the reaction enthalpy (*Q*) dependence function on the total bile salt concentration in the reaction cell (Figure 15).

The pNa values (for the degree of counterion dissociation from the micelle particle) were determined using a Radiometer TitraLab 845 titrator (Hach Lange GmbH, Düsseldorf, Germany) with the ion-selective electrode ISE21Na and the reference electrode RedRod201 (Ag/AgCl) at 25 °C. Calibration was carried out with NaCl solutions [19].

## 4. Conclusions

In the studied 7-alkyl derivatives of cholic acid anions, the critical micellar concentration (cmc) decreases as the number of carbon atoms in the alkyl chain increases, which indicates a rise in hydrophobicity and a greater tendency for self-association at each tested temperature, ranging from 10 to 40 °C. For each derivative analyzed, the relationship between the natural logarithm of cmc and temperature exhibits a U-shaped curve, with the minimum value of ln cmc occurring between 27 and 30 °C. The weak dependence of the standard Gibbs free energy change during demicellization on temperature suggests the presence of enthalpy–entropy compensation, a hallmark of the hydrophobic effect.

In the micelles formed by the tested 7-alkyl derivatives of cholic acid anions, the binding of counter ions is negligible compared to that observed in classic ionic surfactants. This indicates that in the micellar state, the C17 side chains with carboxylate groups are relatively distant from one another.

The change in the standard molar heat capacity during demicellization indicates that when the C7 alkyl chain of a steroid contains more than four carbon atoms, a gauche conformation of the alkyl chain becomes feasible. This conformation does not encounter steric repulsion from the steroid skeleton. Precisely, the alkyl chain can orient itself towards the convex surface of the steroid skeleton, known as the β side. In this arrangement, the hydrophobic hydration, which always occurs in the elongated antiperiplanar conformation of the C7 alkyl chain, is effectively prevented.

## Figures and Tables

**Table 1:** Thermodynamic parameters of micellization of 7-alkyl derivatives of cholic acid anion in aqueous solution: $∆h_{demic}^{0}$ = standard molar enthalpy change in demicellization; $∆g_{demic}^{0}$ = standard change in molar Gibbs free energy of demicellization; $∆s_{demic}^{0}$ = standard molar entropy change in demicellization; $∆C_{demic}^{0}$ = standard molar heat capacity of demicellization; and cmc = critical micellar concentration.

Temperature/°C	$∆h_{demic}^{0}$/kJ mol^−1^	cmc/mM	cmc *10^4^/mol Fraction	$∆g_{demic}^{0}$/kJ mol^−1^	$T∆s_{demic}^{0}$/kJ mol^−1^	$∆C_{demic}^{0}$/JK^−1^ mol^−1^
**C**						
**10**	−4.53	13.50	2.47	19.59	−24.12	253
**15**	−3.81	12.80	2.30	20.07	−23.88	
**20**	−2.29	12.10	2.17	20.56	−22.85	
**25**	−0.90	9.60	1.73	21.47	−22.37	
**30**	0.15	n.d.				
**35**	1.57	15.40	2.77	20.98	−19.42	
**40**	2.89	16.20	2.92	21.18	−18.30	
**7-EthC**						
**10**	−4.71	11.50	2.07	19.96	−24.67	285
**15**	−3.37	11.00	1.98	20.43	−23.79	
**20**	−1.35	10.50	1.89	20.90	−22.25	
**25**	−0.68	9.50	1.71	21.50	−22.18	
**30**	0.55	10.80	1.94	21.54	−20.99	
**35**	2.09	11.30	2.03	21.78	−19.69	
**40**	4.32	12.80	2.30	21.81	−17.49	
**7-ProC**						
**10**	−4.08	10.00	1.80	20.30	−24.38	311
**15**	−3.15	9.80	1.76	20.71	−23.86	
**20**	−1.59	9.20	1.65	21.33	−22.82	
**25**	−0.11	n.d.				
**30**	1.32	9.50	1.71	21.86	−20.54	
**35**	3.75	10.80	1.94	21.90	−18.15	
**40**	4.86	11.50	2.07	22.09	−17.23	
**7-ButC**						
**10**	−5.53	9.60	1.73	20.39	−25.92	321
**15**	−3.93	9.20	1.65	20.86	−24.80	
**20**	−2.75	8.90	1.60	21.30	−24.05	
**25**	−1.59	8.60	1.55	21.74	−23.33	
**30**	0.99	9.10	1.64	21.97	−20.98	
**35**	2.17	9.80	1.76	22.15	−19.98	
**40**	4.14	10.50	1.89	22.32	−18.18	
**7-OctC**						
**10**	−5.97	8.80	1.58	20.61	−26.57	345
**15**	−4.24	8.50	1.53	21.05	−25.28	
**20**	−2.22	8.30	1.49	21.48	−23.70	
**25**	−1.05	7.90	1.42	21.96	−23.01	
**30**	1.25	8.40	1.51	22.17	−20.92	
**35**	2.98	8.90	1.60	22.40	−19.42	
**40**	4.15	9.40	1.69	22.61	−18.46	

The relative standard uncertainty *u_r_* (experimentally directly determined parameters): *u_r_*($∆h_{demic}^{0}$) = 5%, *u_r_*(cmc) = 6%.

**Table 2:** The degree of counterion dissociation from the micelle particle (ϑ) at 25 °C.

7-EthC	7-ProC	7-ButC	7-OctC
0.89	0.93	0.95	0.95

The relative standard uncertainty is *u_r_*(ϑ) = 5%.

## Data Availability

All the relevant data are contained within the paper.
